# Evolving Transplant Oncology: Evolving Criteria for Better Decision-Making

**DOI:** 10.3390/diagnostics15070820

**Published:** 2025-03-24

**Authors:** Filippos F. Karageorgos, Konstantina-Eleni Karakasi, Athanasios Kofinas, Nikolaos Antoniadis, Georgios Katsanos, Georgios Tsoulfas

**Affiliations:** Department of Transplantation Surgery, Center for Research and Innovation in Solid Organ Transplantation, Aristotle University School of Medicine, 54642 Thessaloniki, Greece; filipposk@auth.gr (F.F.K.);

**Keywords:** transplant oncology, selection criteria, biomarkers, liver transplantation, hepatocellular carcinoma, cholangiocarcinoma, clinical decision-making

## Abstract

Transplant oncology integrates a wide variety of fields, such as surgery, oncology, and transplant medicine, intending to increase the range of studies and treatments for hepatobiliary cancers and other liver-related malignant lesions. Liver transplantation (LT) has proven to be an effective treatment for hepatocellular carcinoma. While the Milan criteria are still the gold standard, several new, more inclusive criteria have been proposed, and hepatocellular carcinoma has become a major indication for liver transplantation. The continuous evolution of diagnostic technologies supported this with higher image quality and more accurate staging. This review describes the current applications of transplant oncology in hepatocellular carcinoma, cholangiocarcinoma, neuroendocrine tumors, and liver metastatic disease from colorectal cancer and discusses the path that led to the development of transplant oncology as an organized approach to managing gastrointestinal malignancies through transplantation. More importantly, the significance of a multidisciplinary approach and criteria in the selection of suitable candidates are discussed. In addition, newer aspects of transplant oncology, such as immunotherapy, circulating tumor DNA (ctDNA), novel surgical techniques, and the utilization of artificial intelligence, are presented. Finally, the opportunities and challenges involved in the field’s future, as well as the evolution of the criteria used over the years and insightful thoughts for the future of the criteria, are discussed.

## 1. Introduction

The number of primary liver cancers and liver-metastasized tumors has increased during the previous 50 years [1]. Primary liver cancer ranks fourth globally in terms of cancer-related deaths and is the sixth most common type of cancer diagnosed [1].

### 1.1. Defining Transplant Oncology

The term “transplant oncology” has been originally introduced by Dr. Lerut in 2015 to describe the common ground between liver transplantation and oncologic management [2,3]. A definition of transplant oncology is “cancer treatment and research by means of highly invasive procedures including allo-/auto-transplantation” [4]. Transplant oncology is a relatively new approach to cancer treatment that has promise for the future. This approach entails the combined application of oncology, surgery, and transplant medicine to enhance the treatment and research of primary or secondary liver tumors [4,5,6]. Specifically, in transplant oncology, the malignant liver is completely removed and is replaced by a healthy graft.

### 1.2. Milan Criteria for Hepatocellular Carcinoma and Other Criteria

In 1996, the work of Mazzaferro and his coworkers, liver transplantation regained momentum for the treatment of hepatocellular carcinoma (HCC) in selected patients corresponding within the Milan criteria [7]. These criteria in the following years until recently kept changing worldwide to include more and more patients within the realm of liver transplantation (LT). The criteria include the tumor diameter, number of tumors, total tumor diameter, level of biomarkers, such as preoperative alpha-fetoprotein level, and more. Apart from HCC, other liver-related malignancies are acknowledged as being appropriate for LT, once more under strict guidelines. Perihilar cholangiocarcinoma (PHC), intrahepatic cholangiocarcinoma (ICC), colorectal liver metastases (CRLM), liver metastases from neuroendocrine tumors (NET), and hepatoblastoma are among these cases. The treatment for these patients will be improved by the closer cooperation of multidisciplinary groups (i.e., immunologists, hepatologists, gastroenterologists, transplant oncologists, transplant surgeons, and others). Although the expansion of indications for LT is a lifesaving option for a great number of patients, this action adds a burden and acts as a major limiting factor in the worldwide shortage of available grafts. This is why in early-stage HCC cases, surgical resection or ablation is preferred. In the problem of organ limitation, there are many scientific fields trying to provide solutions with their unique way. Specifically, immunologists are trying to link transplant immunology and tumor immunology to clarify the self and non-self-recognition system. Clinicians are creating new guidelines and protocols for accessing, utilizing, and interlarding liver grafts. Transplant surgeons create novel surgical techniques to increase the number of available grafts. Genomics are utilized to investigate the biomechanism of disease. Even engineers with the futuristic field of organ bioengineering and regenerative medicine try their best for a pragmatic solution to an artificial graft [8], something that in some organs, such as the kidneys, they are closer than ever to achieving [9].

### 1.3. Scope of This Review

The scope of the current review is to seek, in the open literature, LT criteria for HCC and for other liver cancers. These criteria are gathered, presented, and discussed. In addition, future technologies that are proposed to aid in the field of transplant oncology are also presented and discussed. These include immunotherapy, circulating tumor DNA (ctDNA), surgical techniques, and artificial intelligence.

## 2. Methodology

A comprehensive literature search was conducted utilizing two databases (i.e., Scopus and Pubmed). Note that the current review is not systematic [10] or systematized [8]; thus, the PRISMA protocol was not followed. The latest search was conducted on 2 December 2024. The term “Transplant Oncology” was used in both databases. From the results found, a snowballing technique was also used for the selected articles regarding transplant oncology. No specific exclusion criteria were used. However, only relevant articles in transplant oncology were extracted and utilized for this review. From the articles utilized, in the majority they were focusing on/published in the last 5 years, although no automatic filter was implemented in terms of the publication date or any other criterion.

## 3. From Milan Criteria Towards the Future

### 3.1. Hepatocellular Carcinoma

It is known that the incidence ratio of HCC of men to women worldwide is 2.8:1 and there are more 841,000 individuals diagnosed with HCC worldwide [11,12]. Numerous molecular failures, including cell cycle dysregulation, DNA methylation alterations, chromosomal instability, immunomodulation, an increase in HCC stem cells, microRNA dysregulation, and epithelial-to-mesenchymal transition, are all part of the complex pathogenesis of HCC [11,13].

There is an ongoing marathon from 1996 and the Milan criteria for HCC, until today, with the optimization of criteria for LT in HCC patients. The Milan criteria, which still today are regarded as the golden standard, include patients with a solitary tumor with diameter (in cm) ≤ 5 or up to 3 tumors ≤ 3 cm each [7].

The first criteria extension came from the United States of America and especially in the University of California San Francisco (UCSF) where a group of scientists proposed that HCC patients with one lesion that is ≤6.5 cm in diameter or ≤3 lesions with ≤4.5 cm in diameter each, if the total tumor diameter in cm is ≤8, are included for LT [14,15]. Then, the other models came fast from different places around the world. Thus, more groups and transplant societies reformed the original criteria to create a new set of rules allowing patients to be eligible for LT. In the published criteria for HCC are, among others, the Kyoto criteria, the up-to-seven criteria, the Spanish criteria, the Metroticket 2.0 model, the Toronto expanded criteria, the AFP model, and the 5-5-500 criteria [16,17,18,19,20,21,22]. Regarding the survival rate for some of these models, the UCSF had at 5 years at least 72.4% [14]. The Kyoto criteria had a 5-year survival rate at 87% [16]. The up-to-seven criteria had a 5-year overall survival at 71.2% [17]. The Spanish criteria had a 5-year survival rate at 63% [18].

Throughout this evolution, the concept of tumor size (i.e., diameter) and number of lesions, being the sole characteristics in the criteria, changed, including the total diameter. Additionally, alpha-fetoprotein (AFP) was included, and this marked the inclusion of cancer-related biomarkers. In addition to AFP, more biomarkers were included, such as the protein induced by vitamin K absence (PIVKA-II). A collection of criteria for HCC is listed in Table 1.

Another method that is used to incorporate more patients in the current criteria to obtain the benefits of LT is the downstaging of tumors. This could be performed using neoadjuvant treatments, such as loco-regional therapy of HCC lesions, such as transarterial chemoembolization (TACE), ablation, or radiation. In addition, a critical point is the sufficient time gap between downstaging and LT [23]. This is the “ablate and wait” strategy [24].

**Table 1:** Developed criteria used in transplant oncology for hepatocellular carcinoma.

Name	Criteria	Cancer Type	Source
Milan Criteria	A single lesion’s tumor diameter ≤ 5 cm, or, In the case of multiple lesions, no more than 3 tumors, each ≤3 cm, Free of extrahepatic metastases or vascular invasion	HCC	[7]
UCSF Criteria	One lesion with diameter that is ≤6.5 cm or ≤3 lesions, ≤4.5 cm each, if the total tumor diameter in cm is ≤8	HCC	[14,15]
Kyoto Criteria	Tumors up to number ≤ 10 and All with diameter of ≤5 cm and PIVKA-II ≤ 400 mAU/mL	HCC	[16]
Fukuoka Criteria	Not limited in tumor number and size No gross vascular invasion No extrahepatic disease	HCC	[25]
Up-to-seven Criteria	HCCs with 7 as the sum of the number of tumors and The size of the largest tumor (cm)	HCC	[17]
Spanish Criteria	Up to 3 tumors that are ≤5 cm each A cumulative tumor burden ≤ 10 cm	HCC	[18]
AFP model or French Criteria	Tumor size contribution that is 0 (i.e., in lesions ≤ 3 cm), 1 (in lesions 3 cm to 6 cm), and 4 points (i.e., in lesions ≥ 6 cm), Number of lesions contributes 0 points (i.e., ≤3 nodules) and 2 points (i.e., ≥4 nodules) AFP level contribution 0 points (i.e., ≤100 ng/mL), 2 points (i.e., 100–1000 ng/mL), and 3 points (i.e., ≥1000 ng/mL) respectively. Note that low-risk patients had ≤2 total score and high-risk patients had >2 total score	HCC	[19]
Metroticket 2.0 Model	The overall number and size (in cm) of tumors should be ≤7, and AFP at <200 ng/mL The sum of the number and size of tumors should be ≤5, and AFP at 200−400 ng/mL The sum of the number and size of tumors should be ≤4, and AFP at 400−1000 ng/mL	HCC	[20]
Toronto expanded Criteria	They used poor tumor differentiation and cancer-related symptoms to exclude patients likely to have advanced HCC with aggressive biology	HCC	[21]
5-5-500	Diameter of nodule ≤5 cm Nodule number being ≤5 AFP value being ≤500 ng/mL	HCC	[22]

HCC: hepatocellular carcinoma; PIVKA-II: protein induced by vitamin K absence; AFP: alpha-fetoprotein.

### 3.2. Other Liver-Related Malignancies

Concomitantly and apart from HCC, in other liver-related malignancies (i.e., PHC, CRLM, and NET), a list of criteria has been published to provide treatment through LT. A collection of those criteria for PHC, CRLM, and NET is listed in Table 2. For the treatment of NETs, the Milan-NET criteria from 2007 and their revision in 2016 have been published [26]. Also, the Mayo Clinic protocol for perihilar cholangiocarcinoma [27] has been published.

Colorectal cancer (CRC) is one of the most prevalent cancers in the Western world, while being the 4th most common cause of cancer-related death [28]. For the case of CRLM, the selection criteria for CRLM have been proposed by SECA-I and SECA-II studies [29]. At least 25% of individuals with CRC will develop CRLM during their illness, rendering the liver the most frequent location of metastases in patients with CRC [30]. As the third most prevalent cancer in men and the second most frequent cancer diagnosed in women, CRC accounts for 10% of all malignancies diagnosed yearly and 10% of cancer-related deaths globally, posing an immense health care burden. [30,31]. Note that the research is still ongoing and results come continuously [32,33,34]. In a recent publication of Adam et al. [33], the outcome of a study, in patients with permanently unresectable colorectal liver metastases, with two possible treatments, LT plus chemotherapy and chemotherapy only, the researchers concluded that in selected patients, LT plus chemotherapy with organ allocation priority significantly improved survival versus chemotherapy alone [33]. In regions of the world where there are not enough deceased donor allografts to match the demand for transplants, LDLT provides the option for transplant in the context of unresectable CRLM [32].

**Table 2:** Developed criteria used in transplant oncology for various types of liver-related malignancies.

Name	Criteria	Cancer Type	Source
Milan-NET (2007, revised in 2016)	Well-differentiated tumor grade (i.e., G1–G2) A previously resected primary tumor draining through the portal venous system Less than 50% metastatic diffusion of the total liver volume Stable disease/response to therapies for a minimum of six months prior to transplant consideration Absence of extrahepatic illness Patient’s age less than 60 years (this is in relative criteria)	NET	[26]
Mayo Clinic protocol for hilar cholangiocarcinoma	PHC Diagnosis based on a malignant-appearing stricture on cholangiography; malignant endoluminal brushing/biopsy; CA 19-9 greater than 100 U/mL, mass on cross-sectional imaging and/or polysomy on FISH Unresectable disease or arising in PSC Completion of neoadjuvant therapy before the LT Medical suitability for transplant operation	PHC	[27]
Selection criteria for CRLM	No extrahepatic disease Radical resection of the primary and N0 status At least a line of chemotherapy with signs of response using the RECIST 1.1 criteria Favorable tumor biology (i.e., Wild type BRAF, low Oslo score, low Fong score, low CEA, left side primary) PET-FDG for evaluation of tumor metabolic burden Performance status that is characterized as good An interval of at least 6 months between cessation of chemotherapy and transplant operation.	CRLM	[29]

NET: neuroendocrine tumors; PHC: perihilar cholangiocarcinoma; CRLM: colorectal liver metastases; PSC: primary sclerosing cholangitis; FISH: fluorescent in situ hybridization.

### 3.3. Hepatoblastoma

A notable work from 2020 [35] is from the ILTS transplant oncology consensus conference, which entails recommendations for LT for CLRM, liver metastases from NETs, and hepatoblastoma. Note that hepatoblastoma has been becoming more common [28]. Specifically, with a frequency of 1.2–1.5 per million, hepatoblastoma is the most prevalent primary liver cancer in children [28]. Regarding hepatoblastoma, note that liver resection may not always be logistically possible despite chemotherapy, such as in central lesions, which leaves LT as the only viable treatment option with a long-term survival rate free of recurrence [36].

### 3.4. Recurrence After LT

A major important aspect that the novel field of transplant oncology has to overcome is that of recurrence after LT. The recurrence can be high and is calculated at 8–20% of cases [37]. Still under rigorous research is the question of how LT causes tumor recurrence. These reasons include, among others, tumor seeding during hepatectomy, inflammation, and immunosuppression. One promising biomarker to identify minimal residual disease and disease recurrence is liquid biopsy to evaluate ctDNA after transplantation. In addition, recently, the biomarker AFP bound to *Lens culinaris* agglutinin (AFP-L3) and also des-gamma carboxyprothrombin (DCP) have been explored for forecasting early HCC recurrence [38]. Furthermore, the ability of the dynamic α-fetoprotein response (AFP-R) to forecast survival and recurrence outcomes of patients undergoing LT for HCC was examined [39]. The importance of AFP-R in the selection of HCC patients for LT was specifically investigated [39]. This is significant because it allows the safe extension of Milan criteria and other models by incorporating AFP-R into selection criteria enabling LT for patients with acceptable tumor biology features who would not otherwise be considered for a potential cure [39].

## 4. Liquid Biopsy-cfDNA/ctDNA-CTC

As can be found in the open literature biomarkers are “a characteristic that is, objectively measured and evaluated as an indicator of normal biological processes, pathogenic processes, or pharmacologic responses to a therapeutic intervention” [40,41]. Biomarkers, such as cfDNA (cell-free DNA), ctDNA, and immunotherapy are arising tools aiming to contribute to the field of transplant oncology.

### Liquid Biopsy-cfDNA-ctDNA-CTC

**Figure 1:**
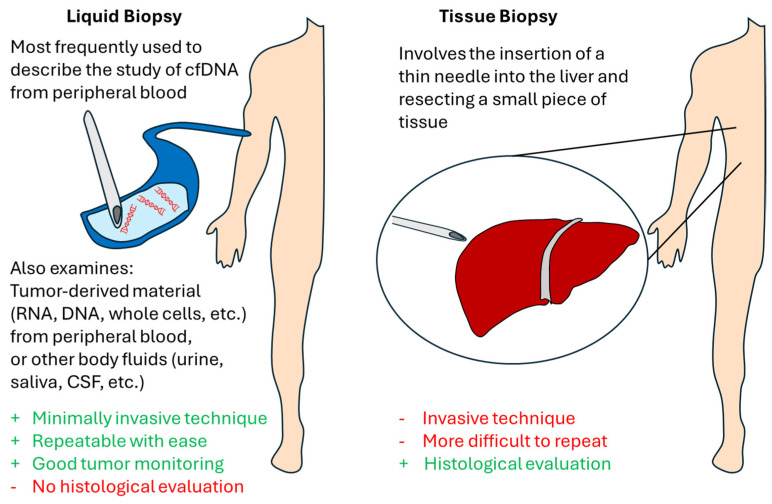
Schematic representation of the comparison of liquid biopsy and liver tissue biopsy.

Although liquid biopsy has most frequently been used to describe the study of cfDNA from peripheral blood, it also refers to the isolation and examination of tumor-derived material from blood or other body fluids, such as RNA, DNA, or even entire cells [42]. Liquid biopsy is a concept that can drastically contribute to monitoring tumor recurrence and organ rejection from LT [43,44,45]. Specifically, liquid biopsy is a minimally invasive technique that commonly uses blood, but other body fluids can also be used (e.g., urine, pleural effusions, ascites, saliva, feces, and cerebrospinal fluid) [46] (please see Figure 1). Liquid biopsy permits repeated analyses in order to monitor tumor metastasis, treatment responses, or recurrence in real time [12]. The cell-derived markers can include, among others, cells (e.g., HCC circulating tumor cells (CTCs)), extracellular vesicles, ctDNA, circulating tumor RNA (ctRNA), microRNA (miRNA), and tumor-educated platelets (TEPs) [12,46]. Note that regarding CTCs, it has been established that circulating cancer tumor cells (CTCs), which originate from the original tumor or metastases, contribute to tumor recurrence [47,48,49,50]. Also, note that the half-life of CTCs is up to 2.4 h, and the detection months or years after primary tumor resection means tumor recurrence or metastasis [12].

Liquid biopsy has both advantages and disadvantages in comparison to solid tissue biopsy. On the one hand, the main advantage of liquid biopsy is its non-invasive nature, as well as the repeatability of the technique that allows for good monitoring of the tumor [46]. On the other hand, liquid biopsy does not allow for a histological evaluation [46]. Assays for cfDNA and CTCs may help to determine patient prognoses and monitor HCC, according to the authors of a recent systematic review that comprised 112 studies that investigated the accuracy of a liquid biopsy analysis [10]. In addition, assays for cfDNA might aid in detection of HCC [10].

It was 1948 when Mandel and Metais first reported cfDNA in blood plasma [42,43]. cfDNA is believed to be released into the bloodstream through apoptosis, necrosis, and secretion [42,43]. It is also typically found as double-stranded fragments of approximately 150–200 base pairs in length [42]. A difference between cfDNA and ctDNA is their structure. Specifically, cfDNA is typically double-stranded DNA, whereas ctDNA is usually shorter in length than non-malignant cfDNA molecules [43]. Note that cfDNA molecules are rapidly cleared from the circulation, with a half-life of an hour or less [42]. The development of blood-based, dynamic biomarkers, including circulating tumor DNA (ctDNA), can assist in identifying the subgroup of patients who are most likely to benefit from adjuvant treatment and in detecting minimum residual illness [51]. Much research has been conducted on the use of ctDNA to assess transplant rejection in non-HCC conditions. New research has also looked into the use of ctDNA detection to assess transplant rejection and the tumor load of HCC in the pre- and post-surgery era [43].

## 5. Immunotherapy

The greatest worry when utilizing immune checkpoint inhibitors (ICIs) peri-transplant is the possibility of graft rejection [52]. ICIs that target either programmed cell death protein 1 (PD-1) or programmed death ligand 1 (PD-L1) have revolutionized the way that HCC is treated and are currently the mainstay of the majority of systemic treatments used in clinical practice and research [53]. As clinical researchers implement and investigate interventive medicines in different combinations and therapeutic situations, the use of immunotherapy has significantly changed the results. Researchers in transplant oncology have been driven to look into whether ICIs can be utilized to bridge more patients with HCC to LT due to the better results seen in these studies utilizing ICIs [54]. Although immunosuppressant use after LT has shown promise in several published trials, there is no concrete proof that immunosuppressants directly prevent or avoid transplant rejection in those who have already received ICIs [54]. Following successful downstaging, that is no extrahepatic disease, an AFP cutoff, and PET non-avid tumor, and response to downstaging therapy to guarantee acceptable tumor biology, patients with proven HCC with macrovascular invasion may be eligible for LDLT [54]. As of right now, there are no agreed-upon guidelines for the use of ICIs to treat HCC in liver transplant recipients [55]. For more information on immunotherapy and LT for HCC, readers are encouraged to read the recent review of some of the authors, which includes more information on downstaging HCC to facilitate liver transplant candidacy [56]. In addition, the recent review of Sankar et al. [53] is also educative in the management of HCC. Moreover, it is of exploratory significance to use chimeric antigen receptor-T (Car-T) treatments in LT for HCC [57]. There are promising prospects for the application of Car-T treatments in immunosuppressive environments [57].

## 6. Other Aspects of Transplant Oncology

### 6.1. Surgical Techniques

Regarding novel transplantation techniques, in the previous decade, the RAPID (resection and partial liver segment 2–3 transplantation with delayed total hepatectomy procedure) and the LD-RAPID (living donor resection and partial liver segment 2–3 transplantation with delayed total hepatectomy procedure) techniques have been developed. These efforts are developed to take into account and treat the liver graft shortage. Particularly, the RAPID technique is the resection and partial liver segment 2–3 transplantation with delayed total hepatectomy [58]. Accordingly, the RAPID technique in living donation is called LD-RAPID [59]. The LD-RAPID technique is the living donor resection and partial liver segment 2–3 transplantation with delayed total hepatectomy [59].

Specifically, by dividing the livers of deceased donors, the RAPID technique creates two grafts (i.e., an extended right and a left lateral graft) that are ready for transplantation, thereby increasing the number of liver grafts available for adult recipients with oncologic indications [28]. As a result, two adult recipients of normal size can receive a hypertrophied left-lateral graft and an extended-right graft through the RAPID technique [27]. The LD-RAPID technique allows for the donation of a smaller section of the liver while still providing enough liver volume for an adult recipient, reducing the risk for the living donor [28].

It is possible to split a liver graft from a deceased donor, a technique that has been used for decades to transplant one adult recipient (with the right part) and one child recipient (with the left part) [60]. However, the possibility of transplanting two adult recipients with one liver graft is rather limited because of the high risk of small-for-size syndrome (SFSS) in the recipient of the left part of the liver graft, which can result in poor outcomes and a frequent need for a retransplant [60].

In order to minimize or at least lessen tumor seeding, the “no-touch recipient hepatectomy” has been implemented in LT to lower the CTC load [47]. When the no-touch total hepatectomy was used, the Chinese and Korean LT teams demonstrated a better clinical outcome, indicating that this strategy might be a powerful way to lower tumor recurrence [47,61,62].

### 6.2. Perfusion Pumps

Furthermore, novel approaches, such as normothermic regional perfusion, normothermic machine perfusion, and hypothermic machine perfusion may improve graft quality and evaluation, which would increase graft-use rates [63]. Donors after circulatory death are a beneficial source of grafts, especially for “marginal” indications, like transplant oncology. They can also be useful assets for expanding the donor pool [63]. With the various types of machine perfusion the chance of successfully transplanting a marginal organ becomes reality [64]. Specifically, machine perfusion is not a new concept, and it is described as follows: organs are attached to a pump and continuously perfused with the solution in a regulated flow until implantation using an ex vivo platform. There are three forms of machine perfusion based on the temperature: normothermic, hypothermic, and subnormothermic [64].

### 6.3. Artificial Intelligence

A set of inputs and a set of outputs make up every facet of organ transplantation, including image processing, result prediction, diagnostic recommendations, therapeutic algorithms, and precision treatments. Artificial intelligence (AI) classifiers vary in their ability to predict the potential alternatives of the output variables, choose which data groups to train patterns on, and create links between the input variables [65]. To accomplish this, there are hundreds of classifiers that can be used. Artificial Neural Networks, Random Forest, Decision Tree classifiers, and Naïve Bayes classification models are the best classifiers to handle the various facets of organ transplantation [65]. The application of AI in organ transplantation is demonstrated in hundreds of instances, particularly in the areas of D-R matching, transplant oncology, precision pathology, organ allocation, image processing, real-time immunosuppression, and predictive analysis [65].

For more precise clinical prediction and data handling, including genetics and imaging in transplant oncology, machine learning (ML) classifiers can be used successfully [66]. This has made it possible to determine the variables that have the biggest effects on survival and recurrence in diseases, like HCC, which aids in predicting which individuals will benefit from a liver transplant [66].

Various groups have employed AI to forecast the oncological outcomes of patients receiving liver transplants for HCC [67]. In a study conducted with 563 patients who underwent LT for HCC in centers in Korea, the researchers aimed to develop a model to predict tumor recurrence after LT by implementing AI, the MoRAL-AI [68]. Tumor diameter was the biggest weighted parameter in the MoRAL-AI, followed by age, PIVKA-II, and AFP [68]. Compared to traditional models, the MoRAL-AI was more accurate in predicting tumor recurrence after LT [68]. Based on preoperative patient and tumor characteristics, the team led by Sapisochin described the application of AI to predict the post-transplant recurrence of HCC [67]. By automatically balancing the concepts of equitable access and optimal usage (utility) in the face of organ scarcity and an expanding waiting list, AI has the potential to greatly improve decision-making [67]. Recently, a prediction model was constructed using AI to predict the risk of post-LT recurrence [69]. The AI model was called Time_Radiological-response_Alpha-fetoproteIN_Artificial-Intelligence (TRAIN-AI) and it combined morphology and biology tumor variables [69]. In total, eight variables were significantly associated with the risk of recurrence and used to construct the TRAIN-AI model [69]. As a result, the model showed better accuracy than other scores for the risk of post-LT HCC recurrence [69]. In a 2021 pilot study, the authors aimed to develop an AI method in order to predict the onset of post-operative sepsis earlier [70]. In the study 5748 transplant and non-transplant individuals were included, with 92 post-liver transplant patients who developed sepsis [70]. In 2020, the work of Ershoff et al. was published [71]. In this work, the authors report that they trained a deep neural network to predict 90-day after-transplant mortality [71]. The results were compared to the Balance of Risk score and Survival Outcomes Following Liver Transplantation score using United Network of Organ Sharing data (*n* = 57,544) on adult patients who were transplanted from 2005 to 2015 from deceased donors [71].

## 7. Discussion

**Figure 2:**
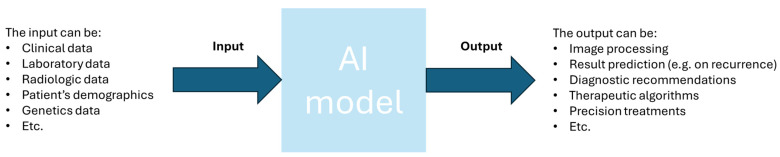
Schematic representation of AI current and possible usage in the field of transplant oncology.

Transplant oncology has enormous potential for treating primary and secondary liver cancers that are inoperable. The multidisciplinary approach of doctors with a range of medical disciplines and other professions is essential to success. These specialties and occupations can include transplant surgeons, pathologists, immunologists, informaticians, and engineers, among others, in order to cooperate in providing the best result in transplantation [4,5,6]. From the current criteria, it is obvious that the newest ones are incorporating not only radiologic findings (i.e., tumor size and number of tumors) but also, they try to incorporate biomarkers in the criteria selection and the biology of the tumors (see criteria in Table 1). The success of the new transplant oncology field is also thought to be aided by meticulous patient selection, cutting-edge targeted medications, novel surgical techniques, and a better knowledge of tumor recurrence processes. Various technologies and innovations can be used, and some of them are used to generate a larger pool for liver grafts. These include the use of surgical techniques, such as RAPID, LD-RAPID, and LDLT [58,59]. Also, these can include the use of machine perfusion and the use of AI. AI especially could be utilized in all of the processes of constructing the criteria, as this technology can aid in the prediction of cancer recurrence, in the classification of the patients, in radiomics, and even more. In the long term, futuristic techniques are believed to be the answer to the primary constraint of transplant oncology, which is a donor shortage (i.e., grafts from the shelf). These include the currently immature solutions, in liver transplantation, fields of regenerative medicine, and organ tissue engineering [8]. These solutions though, as said earlier, are immature, and under the current conditions, no one can actually rely on them or can actually predict the timeline to produce artificial livers. For the time being, the best option for the scientific community is to focus on biomarkers and immunology therapies combined with techniques of AI (see Figure 2), such as supervised learning and unsupervised learning to utilize the current data and try to analyze them, also finding connections that it is possible humans have not yet thought of. Moreover, this has to be performed on the very first steps in national or clinic levels in order to see differences between clinics and nations since the transplant systems in many countries/clinics follow many different rules and procedures. Afterwards, it would be beneficial to examine the data from larger international databases to extract solid conclusions and new criteria that will include thousands of individuals.

## 8. Conclusions

Transplant oncology holds great promise and is continuously under development to ensure better criteria and better procedures for more people. These criteria seem to include more elements than just radiologic findings from the very first Milan criteria, including the biology of the tumor and biomarkers, such as AFP, PIVKA-II, and more. Currently, the Milan criteria dominate in the field, but with the use of AI, new biomarkers, such as ctDNA, and immunology, the criteria will keep evolving to include not only HCC, but also more primary and secondary hepatic tumors.

## Data Availability

No data were generated during this work.
